# Xijiao Dihuang Decoction Alleviates Ischemic Brain Injury in MCAO Rats by Regulating Inflammation, Neurogenesis, and Angiogenesis

**DOI:** 10.1155/2018/5945128

**Published:** 2018-06-25

**Authors:** Xiaojun Fei, Xu Zhang, Qi Wang, Jingbo Li, Hao Shen, Xuanye Wang, Hongquan Liu, Weiwei Tao

**Affiliations:** ^1^Affiliated Hospital of Integrated Traditional Chinese and Western Medicine, Nanjing University of Chinese Medicine, Nanjing 210028, China; ^2^Suzhou Integrated Traditional and Western Medicine Hospital, Suzhou 215101, China; ^3^Nanjing Jiangning Hospital of TCM, Nanjing 211100, China; ^4^Jiangsu Shenlong Pharmaceutical Co., Ltd., Yancheng 224200, China

## Abstract

Ischemic stroke is an increasingly important public health problem, and no effective treatments are approved. Xijiao Dihuang Decoction (XDD), a famous herbal formula for treating hemorrhagic fever syndromes, has been shown to exert powerful neuroprotective property. The aim of this study was to identify the chemical constituents in XDD, observe the neuroprotective effect of XDD against acute ischemic stroke, and explore the specific mechanisms by which these effects were mediated. With UHPLC-Q/TOF-MS, 47 components in XDD were detected and 25 of them were identified. In rats subjected to MCAO, XDD ameliorated neurological deficit, histopathology changes, and infarction volume. In addition, levels of TNF-*ɑ*, IL-6, and IL-1*β* in XDD-treated group were significantly lower compared to the model group. Mechanistic studies showed that XDD inhibited MCAO-induced NF-*κ*B activation, presenting as downregulating the expression of phospho-NF-*κ*B p65 and preventing I*κ*B*ɑ* degradation. Besides, BDNF, GDNF, VEGF, bFGF, and CD34 levels were significantly increased by XDD, suggesting that the protective effects of XDD may also be associated with the promotion of neurogenesis and angiogenesis. In conclusion, these findings provided a novel regulatory pathway of the neuroprotective effect of XDD that helped rehabilitate patients with stroke.

## 1. Background

Ischemic stroke, a major cause of death and neurological disability, is an increasingly important public health problem and imposes a heavy social burden on the family and society. Approximately 15 million people annually experience stroke. About 80 % of strokes are caused by ischemia resulting from an disruption of blood flow followed by induction of hypoxia and glucose deprivation [1, 2]. Cerebral ischemia has complex biochemical and molecular mechanisms involving glutamate-induced excitotoxicity, calcium influx, inflammation response, and blood-brain barrier breakdown [3–5]. At present, the major therapeutic strategies for stroke focus on dissolving clots and restoring blood flow by the administration of thrombolytic drugs such as rt-PA [6]. However, these drugs exhibit serious side effects and very narrow therapeutic time window and thus are only given to a limited group of patients [7]. Furthermore, reperfusion could exacerbate injury in the infarct core and lead to condition known as cerebral ischemia/reperfusion (I/R) injury [8]. Up to now, therapeutic agents that block excitatory neurotransmission, prevent the ischemic inflammatory response, or scavenge free radicals have been evaluated and showed promising therapeutic potential in animal stroke models [9, 10]. However, no effective treatments that can reduce stroke size or neurological disability in humans are approved.

Traditional Chinese medicine (TCM) has been practiced in China for more than 2000 years. Its application in treating stroke-like symptoms has a long history. The herbal medicine therapy has been regarded as an alternative and promising strategy for the treatment of ischemic stroke [11]. Xijiao Dihuang Decoction (XDD) is a famous ancient Chinese formula recorded in “Prescriptions Worth A Thousand Gold”, traditionally prescribed for stopping bleeding accompanied with fever, removing toxic substances, and treating spontaneous bleeding, hemoptysis, and nosebleeds [12]. XDD is composed of aqueous extract of Bubali Cornu (water buffalo horn), Radix Rehmanniae (the dried root of Rehmannia glutinosa (Gaertn.) DC.), Radix Paeoniae Alba (the dried rhizome of Paeonia lactiflora Pall), and Moutan Cortex (the root bark of Paeonia suffruticosa Andr.) at a ratio of 10:8:4:3. All the herbs in XDD are officially listed in the Pharmacopoeia of PR China (edition 2015). Under the guidance of TCM theory, Bubali Cornu is the principal medicinal that can cool the blood for hemostasis and remove toxic substances. Radix Rehmanniae is the ministerial medicinal with activities of heat-clearing and yin-nourishing. Radix Paeoniae Alba and Moutan Cortex are used to invigorate the circulation of scattered stasis symptoms [13]. Recently, an increasing amount of evidence has revealed the pharmacological effects of XDD on inflammation, oxidative damage, virus infection, acute liver injury, and rheumatoid, as well as in lupus erythematosus [14–16]. More specifically, studies on the pharmacological and biochemical activities of XDD extract and its constituents have also showed that it has protective effects against ischemic stroke. For example, Liu et al. [17] showed that XDD posttreatment could reduce ischemia-reperfusion brain injury and autophagy in rats. Lu et al. [18] indicated that XDD could improve neurological function deficit in subjects with acute cerebral hemorrhage. Pretreatment with catalpol and paeoniflorin, two major bioactive components of XDD, suggests beneficial effects on improving the stroke index, increasing SOD activity, decreasing the brain MDA content, and reducing cerebral infarct and neurological deficit in ischemia-reperfusion injured rats [19, 20]. Despite the evidence that XDD is an attractive candidate for neuroprotective agent in the treatment of stroke, the mechanism of pharmacological action of XDD is still unclear.

In this work, the effects of XDD on ischemic brain injury, as well as its actions on inflammation, angiogenesis, and neurogenesis in a rat model of cerebral I/R injury were investigated. This is the first study, to our knowledge, to examine the potential link between the XDD and neurogenesis or angiogenesis in response to cerebral ischemia/ reperfusion.

## 2. Methods

### 2.1. Chemical and Reagents

Nimodipine (Nim) was obtained from Bayer pharmaceutical Co., Ltd (Leverkusen, German). Antibodies recognizing NF-*κ*B p65, I*κ*B*ɑ*, BDNF, GDNF, VEGF, bFGF, CD34, *β*-tubulin, and GAPDH were supplied by Santa Cruz Biotechnology, Inc. Secondary antibodies were obtained from Shanghai Beyotime Biotechnology Co., Ltd. ELISA Kits for TNF-*ɑ*, IL-6, and IL-1*β* detection were purchased from Shanghai Enzyme-Linked Biotechnology Co., Ltd.

### 2.2. Preparation of XDD

XDD was composed of 4 kinds of crude drug, each of which was purchased from Jiangsu Hospital of Integrated Traditional Chinese and Western Medicine (Jiangsu, China). The voucher specimens of Bubali Cornu (water buffalo horn), Radix Rehmanniae (the dried root of Rehmannia glutinosa (Gaertn.) DC.), Radix Paeoniae Alba (the dried rhizome of Paeonia lactiflora Pall), and Moutan Cortex (the root bark of Paeonia suffruticosa Andr.) were deposited in the laboratory of Cellular and Molecular Biology at Jiangsu Province Academy of Traditional Chinese Medicine. The preparation of XDD is described as follows: 30g Bubali Cornu, 24g Radix Rehmanniae, 12 g Radix Paeoniae Alba, and 9g Moutan Cortex were refluxed twice with water (1:10 for the first extraction and 1:8 for the second extraction, w/v) for 2 h and filtered. The filtrates were concentrated and evaporated under vacuum to yield the extract solution (3g/mL).

### 2.3. UPLC-Q/TOF-MS Analysis

A Waters ACQUITY UPLC™ system together with a Q-TOF Synapt G2 mass spectrometer (Waters Corporation, Milford, MA, USA) was used for UHPLC-Q/TOF-MS analysis. Chromatographic separations were performed on a Waters ACQUITY HSS T3 column (100 mm×2.1 mm, 1.8 *μ*m). The mobile phase consisted of 0.1% formic acid (A) and acetonitrile containing 0.1% formic acid (B). The gradient eluted conditions were as follows: 0–15 min, 5–50% B; 15–17 min, 50–95% B; 17–21 min, 95% B; and the posttime was 6 min for column equilibration. The flow rate was set to 0.3 mL/min and the injection volume was 3 uL. The autosampler was maintained at 15°C. An electrospray ionization source was used in negative ion mode. The accurate mass and elemental composition for the precursor ions and fragment ions were analyzed by the MassLynx V4.1 software (Waters Co., Milford, USA).

### 2.4. Animals Approval and Stroke Induction

All experimental procedures were approved by Nanjing University of Chinese Medicine and were conducted according to the Guide for the Care and Use of Laboratory Animal of the National Institute of Health (Publication No. 80–23, revised 1996). Attempts were made to minimize suffering and reduce the numbers of animals used.

Male Sprague-Dawley rats weighing 250-280g were used and housed in the same animal care facility during a 12 h light/dark cycle. The MACO model was established by transient middle cerebral artery occlusion as mentioned earlier, as previously described [21]. In short, rats were anesthetized by intraperitoneal injection of 10% chloral hydrate (350 mg/kg) and placed on heating pad to maintain rectal temperature at 37°C. Then, the right common carotid artery (CCA), external carotid artery (ECA), and internal carotid artery (ICA) were exposed through a midline ventral incision. A nylon filament was inserted from the left ECA into the lumen of the ICA to occlude the origin of the middle cerebral artery (MCA) for 2 h, and the filament was subsequently withdrawn for blood reperfusion. The sham group underwent identical procedure described above, without the MCA occlusion.

### 2.5. Drug Treatment

Before the surgical operation, the male SD rats were randomly divided into 5 groups (10 animals / group): the sham group, the MACO model group, XDD-H (22.5 g/kg) group, XDD-L (11.25 g/kg) group, and the Nim (10 mg/kg) group. From days 1 to 7 before MACO and 5 days after MACO, rats in XDD and Nim groups were orally given their respective dose once daily. The sham and MCAO controls were given an equal volume of the vehicle (0.9% physiological saline).

### 2.6. Neurological Assessment

Neurological deficits were evaluated according to Longa's methods 24 h after reperfusion [22]. The criteria were set as follows: 0, no deficit; 1, failure to fully extend the left forepaw; 2, circling to the left; 3, falling to the left; 4, no spontaneous walking with a depressed level of consciousness. The rats with scores between 2 and 4 were selected for the present study.

### 2.7. Measurement of Infarct Volume

Twenty-four hours after reperfusion the rats were sacrificed following anesthetization. The brains were removed, immediately frozen in liquid nitrogen, and dissected into coronal sections (2 mm thick, n=6 for each group). Subsequently, samples were immersed in 0.1% TTC solution at 37°C for 30 min. The noninfarcted areas were stained to be red-purple, whereas infarcted parts were white. Slices stained were photographed, and infarct size was calculated by Image-Pro Plus image processing software, expressed as infarct area percentage (%).

### 2.8. TTC Staining

The fresh brain tissues of rats after 24 h reperfusion were excised and immediately frozen at -20°C for 20 min. The brain tissues were then sectioned along the coronal plane into and then sliced into 5 uniform 2-mm sections. The central three sections were incubated in 2,3,5-triphenyltetrazolium chloride (TTC) for 30 min at 37°C. Slices stained were photographed, and infarct size was calculated as infarct area percentage (%).

### 2.9. Hematoxylin and Eosin (HE) Staining

The brains were fixed in 4% paraformaldehyde and embedded in paraffin to be cut into 5 *μ*m sections. Then, HE staining was performed with 0.5% hematoxylin (Sinopharm Chemical Reagent Co., Ltd) according to the manufacturer's instructions. The staining images were acquired using a light microscope with 200× magnification.

### 2.10. Measurement of TNF-*ɑ*, IL-6, and IL-1*β*

The penumbras in ipsilateral ischemic brain hemisphere (right brain) samples of the different treatment groups were rinsed, homogenized in PBS, and stored overnight at -20°C. After two freeze-thaw cycles, the homogenates were centrifuged, and the supernatant was removed and assayed immediately. Protein content was assayed by the BCA procedure. The levels of TNF-*ɑ*, IL-6, and IL-1*β* were measured using commercially available quantitative ELISA kits (n=10 in each group). The change in absorbance was measured at 450 nm with a spectrophotometer. Values of cytokine levels were expressed in terms of milligrams per gram (mg/g).

### 2.11. Western Blotting

Equal amounts (20~40 *μ*g) of protein prepared from the penumbra in ipsilateral ischemic brain hemisphere were subjected to sodium dodecyl sulfate-polyacrylamide gel electrophoresis (SDS-PAGE) with electrophoretic transfer onto PVDF membranes. After blocking with 5 % (w/v) BSA, membranes were incubated separately with corresponding primary antibodies (dilution 1  : 1000) overnight at 4°C. After complete washing with TBS-T, membranes were incubated with peroxidase-conjugated goat anti-rabbit antibodies at a dilution of 1:2000 for 2 h at room temperature. The bands corresponding to the interested proteins were visualized by enhanced chemiluminescence. Images were analyzed by using Bio-5000 plus scanner provided by Microtek International, Inc. The protein expression was normalized to the *β*-tubulin or GAPDH protein levels (n = 3 in each group).

### 2.12. Statistical Analysis

For statistical analysis, the GraphPad Prism 5.0 software package was used (GraphPad Software, Inc., La Jolla, CA, USA). All data were presented as mean ± standard error of the mean (SEM). One-way analysis of variance (ANOVA) followed by post hoc Tukey's test for multiple comparisons was used. P < 0.05 was considered to be significant.

## 3. Results

### 3.1. Phytochemical Analysis of XDD by UPLC-Q/TOF-MS

**Figure 1:**
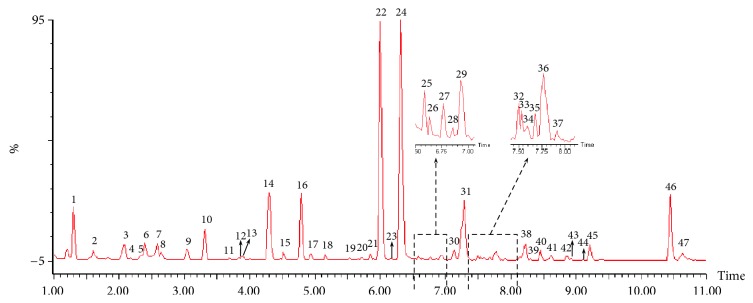
Representative base peak intensity (BPI) chromatograms of XDD analyzed by LC-MS.

In this study, UPLC-Q/TOF-MS analysis was conducted on XDD to confirm its biological composition. Consequently, a total of 47 components were detected and 25 of them were identified, with the identity of 5 compounds confirmed by reference compounds. The constituents were tentatively assigned by matching the empirical molecular formula of each constituent with those of the published compounds and/or by elucidating the quasi-molecular ions and fragment ions. Typical base peak intensity (BPI) chromatogram of the XDD is presented in Figure 1, with the properties of identified compounds summarized in Table 1.

### 3.2. XDD Pretreatment Reduces Functional Deficit and Ischemic Injury in MCAO Rats

**Figure 2:**
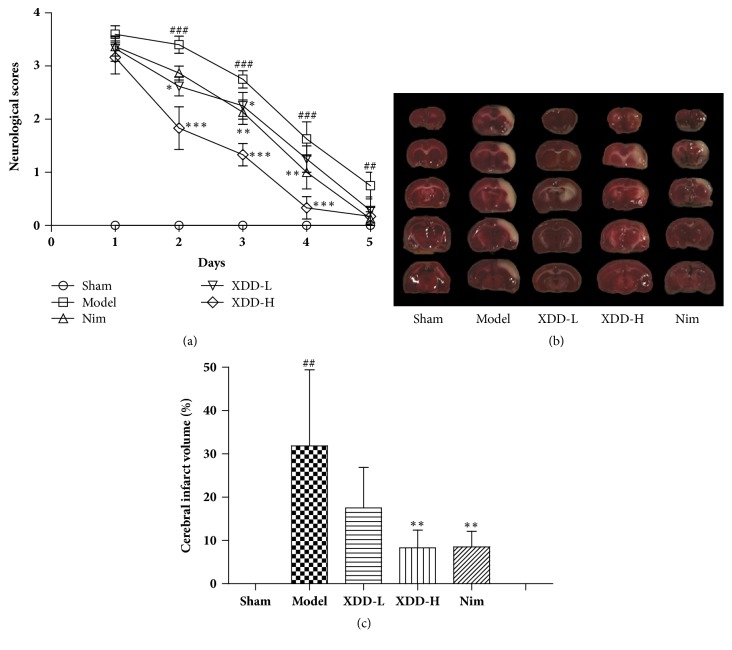
Neuroprotective effects of XDD, denoted by neurological deficit scores (n=10) and TTC staining. (a) Effects of XDD on neurological deficit scores (n=10) of rats subjected to 2 h MCAO and 5 days of reperfusion injury. (b) Effects of XDD on infarct regions (white) in TTC-stained rat brain sections after MCAO/R injury. (c) Statistical analyses of the effects produced by XDD on infarct volume in TTC-stained sections after MCAO/R injury (n=6). ^##^P < 0.01 and ^###^P < 0.01 versus sham; ^*∗*^P < 0.05, ^*∗∗*^P < 0.01, and ^*∗∗∗*^P < 0.05 versus model.

We explored whether XDD is neuroprotective against acute ischemic injury by using the rat transient MCAO model (2 h occlusion followed by reperfusion) and a pretreatment regimen. As shown in Figure 2(a), no neurological symptoms were observed in any rats with sham operation. MCAO strongly affected neural behavior as indicated by the neurological scores of the model group measured at days 1 (F=952.2, P<0.001), 2 (F=1156, P<0.001), and 3 (F=756.25, P=0.001) after MCAO (score >2). The rats pretreated with XDD showed a dose-dependent decreased neurological deficit score, and the improvements were significant at days 2 (all P<0.05 or P<0.001) and 3 (all P<0.01 or P<0.001) after MCAO. The neurological deficit score of rats treated with XDD at dose of 22.5 g/kg was lower than that of rats given Nim (10 mg/kg, Figure 2(a)).

### 3.3. XDD Pretreatment Reduces Infarction Volume in MCAO Rats

Infarction volume in MCAO rats was evaluated by TTC staining and imaging software. Representative samples of TTC-stained brain sections are shown in Figure 2(b), with corresponding infarction volumes and statistical data that are shown in Figure 2(c). None of the rats in the sham group exhibited infarction volume. In contrast, infarct volume was significantly increased in the model group. About 31.83±7.85% of the area of the right hemisphere in MCAO model group rats could not be stained red (F=13.74, P=0.0052). Pretreatment with XDD could decrease the infarction volume of MCAO rats. XDD at dose of 22.5g/kg reduced the infarct volumes induced by I/R to 8.33±4.08% (F=13.74, P=0.0039), with the inhibition rate being 73.82% (XDD at dose of 22.5g/kg versus model group). For the positive control group, Nim at a dose of 10 mg/kg also significantly reduced I/R-induced infarct volume (F=24.66, P=0.0011).

### 3.4. XDD Pretreatment Ameliorates Histopathological Alteration in Brains of MCAO Rats

**Figure 3:**
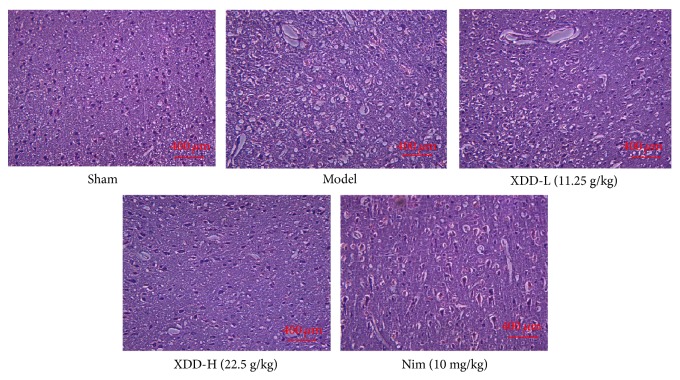
Degeneration of brain tissues and neurons in I/R group, ameliorated by XDD pretreatment (magnification ×200).

As shown in Figure 3, the cerebral tissues and cells in MCAO model group rats were severely damaged: Brain tissue was observed as an appearance of liquefied changes and polynesic sponginess, and glial cells exhibited swelled degeneration, with neurons being in a disordered arrangement, and shrinking and dark staining in nuclei. XDD of high dosage significantly relieved the abnormalities caused by ischemia/reperfusion. No significant difference from the model control group was found for the high dose XDD or Nim-treated groups, while pathological abnormalities were still generally observed for low dose of XDD group.

### 3.5. XDD Pretreatment Reduces Levels of TNF-*ɑ*, IL-6, and IL-1*β* in MCAO Rats

**Figure 4:**
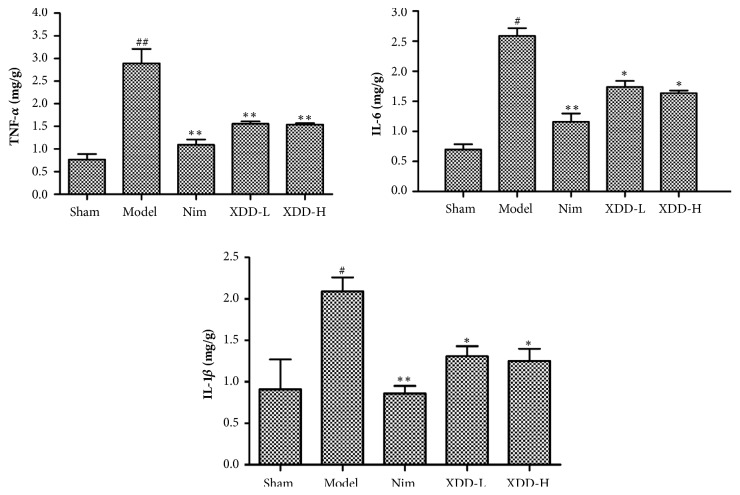
Effects of XDD on the protein levels of the inflammatory mediators TNF-*ɑ*, IL-1*β*, and IL-6 in rat brains after MCAO/R injury (n=5, ^#^P < 0.05, ^##^P < 0.01 versus sham; ^*∗*^P < 0.01, ^*∗∗*^P < 0.001 versus model).

Inflammatory cytokines such as TNF-*α*, IL-1*β*, and IL-6 play a role in the induction and pathoprogression of stroke. To explore the effects of XDD on the inflammatory process in the postischemic brain, we examined levels of cytokines in the ischemic regions using ELISA. After MCAO, levels of TNF-*ɑ*, IL-6, and IL-1*β* were significantly elevated in model group rats compared to sham group rats (for TNF-*ɑ*, F=103.08, P =0.0025; IL-6, F=27.47, P =0.0345; IL-1*β*, F=69.62, P =0.0140; Figure 4). But in XDD-treated group levels of TNF-*ɑ*, IL-6, and IL-1*β* were all significantly lower compared to the model group (P < 0.05, Figure 4). These findings suggested that XDD could suppress brain inflammation responses after stroke.

### 3.6. XDD Inhibited MCAO-Induced NF-*κ*B Activation

**Figure 5:**
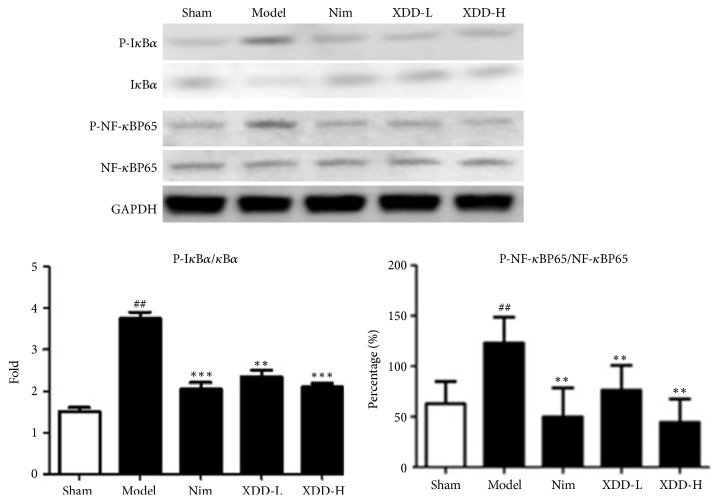
Effects of XDD on the protein levels of NF-kB p65 and IkB*α* in rat brains after MCAO/R injury (n=3, ^##^P < 0.001 versus sham; ^*∗∗*^P < 0.01, ^*∗∗∗*^P < 0.001 versus model).

To explore the mechanisms underlying the anti-inflammation effect of XDD, NF-*κ*B activation was investigated. First, the NF-*κ*B p65 subunit expression was detected after MCAO. The results showed that phospho-NF-*κ*B p65 was upregulated (P < 0.01), whereas NF-*κ*B p65 showed no change (Figure 5), suggesting that cerebral ischemia activated NF-*κ*B signaling. XDD treatment significantly inhibited the expression of phospho-NF-*κ*B p65 (P < 0.01). Degradation of I*κ*B proteins is an essential step in activation of NF-*κ*B. Therefore, we further studied the effect of XDD on the MCAO-induced I*κ*B*ɑ* degradation. Western blotting analysis indicated that the phosphorylation level of I*κ*B*ɑ* was significantly upregulated and the expression of I*κ*B*ɑ* was decreased after MCAO (P < 0.01). XDD markedly blocked I*κ*B*ɑ* phosphorylation and prevented I*κ*B*ɑ* degradation (Figure 5, P < 0.001) in the ischemic penumbra of the brain.

### 3.7. XDD Pretreatment Enhanced BDNF and GDNF Expressions in the MCAO Rats

**Figure 6:**
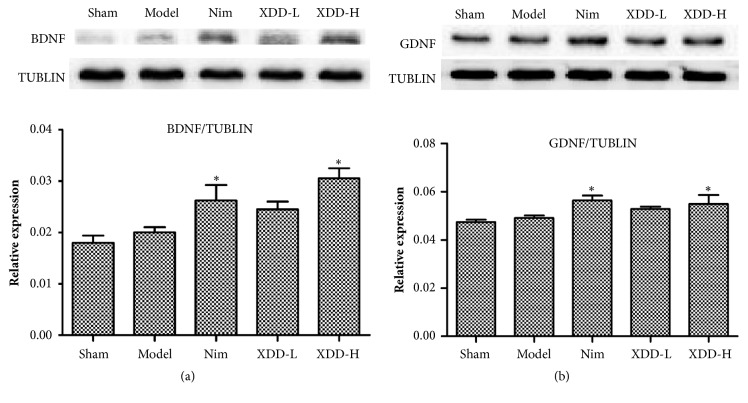
Effects of XDD on the expression levels of BDNF (a) and GDNF (b) in rat brains after MCAO/R injury (n=3, ^*∗*^P < 0.05 versus model).

Brain derived neurotrophic factor (BDNF) is one of the neurotrophic factors that related to neurogenesis. In our work, the expression of BDNF was enhanced after I/R injury as compared with the sham group (Figure 6(a)). Before I/R injury, the rats were treated with different doses of XDD (11.25 or 22.5g/kg) for 7 days. The results showed that the BDNF expressions were significantly higher in the 22.5g/kg XDD treatment group and Nim-treated group compared with the model group (P<0.05; Figure 6(b)). The findings suggested that XDD strengthened the expression of BDNF and promoted neurogenesis after ischemia/reperfusion. Glial cell line-derived neurotrophic factor (GDNF), a potent neurotrophic factor promoting neuronal survival and differentiation, has been shown to have therapeutic effect on ischemic brain injury [23]. In this study, the effects of XDD on the expression of GDNF were analyzed. After a 2 h ischemic insult followed by reperfusion, the expression level of GDNF was elevated in the model group. Treatment with XDD (22.5g/kg) and Nim (1 mg/kg) significantly (P<0.05) further increased the GDNF protein expression compared with sham group or MCAO rats treated with vehicle (Figure 6(b)). These results demonstrate that XDD may promote neurogenesis after cerebral ischemia-reperfusion in rats.

### 3.8. XDD Pretreatment Enhanced VEGF, bFGF, and CD34 Expressions in the MCAO Rats

**Figure 7:**
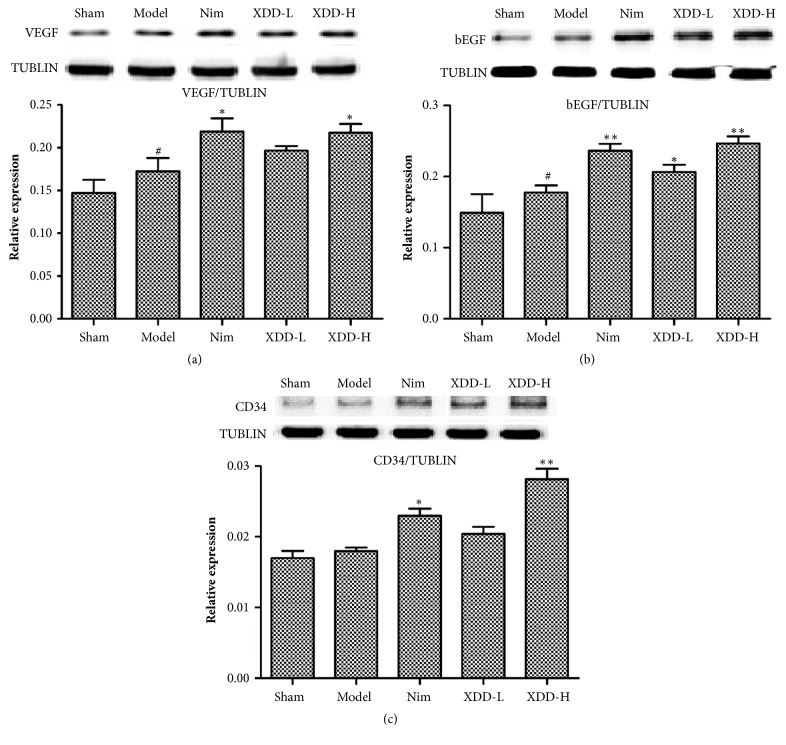
Effects of XDD on the protein levels of VEGF (a), bFGF (b), and CD34 (c) in rat brains after MCAO/R injury (n=3, ^#^P < 0.05 versus sham; ^*∗*^P < 0.05 versus model, ^*∗∗*^P < 0.01 versus model).

Appropriate restoration of blood flow via angiogenesis is a key process for the recovery from ischemic neuronal injury. Vascular endothelial growth factor (VEGF), known as a key factor favoring angiogenesis, has been shown to exert neuroprotection against a wide variety of insults, including ischemic neuronal injury. Basic fibroblast growth factor (bFGF) is also an important angiogenic factor that facilitates angiogenesis. Several studies have reported the upregulation of bFGF expression after injury or ischemia to the mature rodent brain. In addition to VEGF and bFGF, the CD34 is an endothelial antigen that has been used as a direct marker of the degree of neoangiogenesis. In the present study, we tested whether XDD enhances postischemic cerebral angiogenesis by measuring the expressions of VEGF and bFGF as well as CD34. The western blotting analysis of ischemic regions suggested the VEGF, bFGF, and CD34 protein expressions increased after modeling. The expression of VEGF and bFGF in the model group was significantly higher than those in the sham control group (Figures 7(a) and 7(b), P < 0.05). Pretreatment with XDD at doses of 22.5 g/kg and 11.25 g/kg could obviously improve levels of VEGF, bFGF, and CD34 compared with model group (P < 0.05 or P < 0.01; Figures 7(a), 7(b), and 7(c)). Although VEGF expression in Nim-treated group was higher than that in XDD-treated group (22.5g/kg), there was no statistical significance between the two groups. These data indicated that XDD might promote angiogenesis via upregulating the VEGF, bFGF, and CD34 protein expressions, which may be one of the mechanisms for inhibiting the cerebral ischemia-reperfusion injury.

## 4. Discussion

Stroke is one of the leading causes of morbidity and mortality in humans. Many chemical drugs such as NMDA receptor antagonist, calcium antagonists, and pituitary adenylate cyclase-activating polypeptide (PACAP) show neuroprotective effects for stroke treatment [24, 25]. Nevertheless, side effects such as cerebral hemorrhage, gastrointestinal irritation, and resistance to drugs may exceed their clinical benefits for long-term application. Nowadays, with advantage of minimal side effects and multidirectional regulations, traditional Chinese medicine (TCM) preparations have become more and more popular in ischemic stroke therapy [26]. The theory of TCM has unique advantages in “preventive treatment of disease”. In the present study, we first identified 25 compounds in XDD by UPLC-Q/TOF-MS and then demonstrated that XDD could alleviate ischemic brain injury induced by MCAO. MCAO-induced focal cerebral I/R injury was the animal model which is most frequently used because its pathological process was very similar to that of clinical cerebral apoplexy. For MACO model, as the infarct is complete by 24 hours and even earlier in the striatum, even the most efficacious neuroprotective therapies are unlikely to show any efficacy if given after this point [27]. Therefore, XDD was chosen to be given to rats before and after MCAO in this study, with a purpose of providing the best protection against ischemic brain injury. By developing focal cerebral I/R injury in rats, we found that pretreatment with XDD for 7 days followed by I/R significantly attenuated I/R-induced disability and histological damage and reduced the secretion of TNF-*α*, IL-6, and IL-1*β*. Mechanistic studies showed that the neuroprotective effect of XDD was related to inhibition of NF-*κ*B activation and upregulation of neurogenesis (BDNF, GDNF) and angiogenesis factors (VEGF, bFGF, and CD34). These results potentially explain the preventive effect of XDD against ischemic brain injury in clinic practice.

Although the pathogenesis of ischemic stroke and its mechanism is complex, increasing evidence has demonstrated that inflammatory responses are predominantly involved in the pathogenic progression of stroke [28]. Inflammatory process is involved in all stages of the ischemic cascade, from the early damaging events to the late regenerative processes. After the onset of ischemia, inflammation exacerbates delayed infarct expansion, and tissue injury begins with an inflammatory reaction [29]. Inflammatory mediators such as IL-1*β*, IL-6, TNF-*ɑ*, iNOS, and COX-2 are upregulated following cerebral I/R surgery [30]. Elevated levels of inflammatory mediators may exacerbate neurological damage by activating various downstream pathways. Targeting one or all of these factors may present potential for preventing ischemic brain tissue damage. In the present study, our results showed that XDD at 11.25 or 22.5g/kg significantly decreased the levels of TNF-*α*, IL-1*β*, and IL-6 in the penumbra of MCAO rats, indicating that XDD decreased neuroinflammation after I/R injury. In addition, XDD markedly decreased the phosphorylation of NF-*κ*B p65 and I*κ*B*α*. These results showed that NF-*κ*B signaling pathway may be involved in the neuroprotective action of XDD.

The present study also demonstrated that promotion of neurogenesis and angiogenesis may be mechanism by which XDD exerted neuroprotection against cerebral I/R injury. Accumulating studies in various experimental models suggest that ischemic brain insults potently stimulate progenitor proliferation. Neuron progenitors are able to migrate to injury sites and act as a part of endogenous repair response after stroke. Pharmacological interventions related to neurogenesis have been considered as a critical strategy to improve the neuron functions after cerebral I/R injury. The neurotrophins (BDNF and GDNF) have recently emerged as an important regulator of adult neurogenesis [31]. BDNF and GDNF stimulate neurogenesis and enhance the appearance and migration of new neurons in the subventricular zone and dentate gyrus [32]. Postischemic intravenous BDNF treatment presents beneficial effect on narrowing infarct size, protecting penumbral neurons, and repairing broken neurons [33]. In the present study, with regard to BDNF and GDNF, cerebral ischemia increased its protein expression levels, which is consistent with a previous study that reported elevated BDNF and GDNF protein expression in transient cerebral ischemia [34]. Treatment with XDD further increased the levels of BDNF and GDNF, and the difference was significant between the XDD-treated group and model control group. Similar to neurogenesis, angiogenesis may also contribute to the functional recovery after stroke. Within minutes of the onset of cerebral ischemia, proangiogenic genes are upregulated and participate in neurovascular remodeling and recovery. Various growth factors have proved to stimulate angiogenesis, including VEGF, bFGF, TGF-*ɑ*, and PDGF [35]. Among them, VEGF is the most potent angiogenic factor with angiogenic, mitogenic, and vascular permeability enhancing activity in endothelial cells. Moreover, several studies have reported that VEGF overexpression reduces infarct volume, improves postischemic motor function, and contributes to brain recovery and repair [36]. The CD34 is an endothelial antigen that has been used to highlight the microvessel density as a direct marker of the degree of neoangiogenesis [37]. In the present study, the effect of XDD on angiogenesis was investigated. The results showed that VEGF, bFGF, and CD34 levels were significantly increased by XDD treatment, suggesting that the protective effects of XDD may be associated with the promotion of angiogenesis.

## 5. Conclusion

This is the first study where 25 kinds of chemical composition in XDD were identified by UPLC-Q/TOF-MS. Subsequently this study shows that XDD was able to ameliorate cerebral I/R injury, and its protective effects may be induced by inhibiting IR-induced upregulation of the inflammatory cytokines IL-1*β*, IL-6, and TNF-*ɑ* via preventing NF-*κ*B activation and by promoting the expression of the neurogenesis-related factors (BDNF and GDNF) and angiogenesis-related factors (VEGF, bFGF, and CD34) in the ischemic brain. Even though the findings of this study are limited and preliminary, they provide a novel regulatory pathway of the neuroprotective effects of XDD that help rehabilitate patients with stroke in clinical practice.

## Figures and Tables

**Table 1:** The identified phytochemical compounds in Xijiao Dihuang Decoction (XDD) detected by UHPLC-MS.

Peak no.	Identity	t_R_(min)	Mean measure mass (Da)	Theoretical exact mass (Da)	Mass accuracy (ppm)	Empirical formula and proposed adduct ions or fragment ions
^*∗*^1	catalpol	1.30	407.1194	407.1190	1.0	C_16_H_23_O_12_[M-H+HCOOH]^−^
			361.1136	361.1135	0.3	C_15_H_21_O_10_[M-H]^−^
2	1′-O-galloylsucrose, 6′-O-galloylsucrose and 6-O-galloylsucrose	1.60	493.1207	493.1193	2.8	C_19_H_25_O_15_[M-H]^−^
3	Gallic acid	2.07	169.0140	169.0137	1.8	C_7_H_5_O_5_[M-H]^−^
6	1′-O-galloylsucrose, 6′-O-galloylsucrose and 6-O-galloylsucrose	2.39	493.1194	493.1193	0.2	C_19_H_25_O_15_[M-H]^−^
7	1′-O-galloylsucrose, 6′-O-galloylsucrose and 6-O-galloylsucrose	2.58	493.1191	493.1193	-0.4	C_19_H_25_O_15_[M-H]^−^
9	Oxypaeoflorin sulfonate	3.04	559.1113	559.1122	-1.6	C_23_H_27_O_14_S[M-H]^−^
10	Lamiidol	3.32	393.1396	393.1397	-0.3	C_16_H_25_O_11_[M-H]^−^
^*∗*^14	Paeoniflorin sulfonate	4.29	543.1177	543.1197	-3.7	C_19_H_27_O_18_[M-H]^−^
15	Oxypaeoflorin isomer	4.51	495.1501	495.1503	-0.4	C_23_H_27_O_12_[M-H]^−^
^*∗*^16	Oxypaeoflorin	4.79	495.1500	495.1503	-0.6	C_23_H_27_O_12_[M-H]^−^
17	(+)-Catechin	4.94	289.0712	289.0712	0.0	C_15_H_13_O_6_[M-H]^−^
18	Mudanpioside E	5.15	525.1607	525.1608	-0.2	C_24_H_29_O_13_[M-H]^−^
21	Purpureaside C/Echinacoside	5.84	785.2502	785.2504	-0.3	C_35_H_45_O_20_[M-H]^−^
^*∗*^22	Albiflorin	6.00	479.1556	479.1553	0.6	C_23_H_27_O_11_[M-H]^−^
			525.1621	525.1608	2.5	C_24_H_29_O_13_[M-H+HCOOH]^−^
^*∗*^24	Paeoniflorin	6.31	479.1549	479.1553	-0.8	C_23_H_27_O_11_[M-H]^−^
			525.1616	525.1608	1.5	C_24_H_29_O_13_[M-H+HCOOH]^−^
30	Isoacteoside/Acteoside/Forsythoside A	7.13	623.1976	623.1976	0.0	C_29_H_35_O_15_[M-H]^−^
31	Galloyl paeoniflorin/Galloylabiflorin/their isomer	7.28	631.1662	631.1663	-0.2	C_30_H_31_O_15_[M-H]^−^
35	iso-Mudanpioside E	7.68	615.1703	615.1714	-1.8	C_30_H_31_O_14_[M-H]^−^
36	Galloyl paeoniflorin/Galloylabiflorin/their isomer	7.77	631.1653	631.1663	-1.6	C_30_H_31_O_15_[M-H]^−^
38	Isopaeoniflorin or Albiflorin R1	8.23	479.1546	479.1553	-1.5	C_23_H_27_O_11_[M-H]^−^
41	unknown	8.62	507.1490	507.1503	-2.6	C_24_H_27_O_12_[M-H]^−^
42	Benzoyloxypaeoflorin	8.84	599.1762	599.1765	-0.5	C_30_H_31_O_13_[M-H]^−^
45	Mudanoside C	9.21	599.1779	599.1765	2.3	C_30_H_31_O_13_[M-H]^−^
46	Benzoyl paeoniflorin	10.45	629.1872	629.1870	0.3	C_30_H_31_O_13_[M-H]^−^
47	Benzoyl paeoniflorin isomer	10.63	629.1866	629.1870	-0.6	C_30_H_31_O_13_[M-H]^−^

## Data Availability

The data used to support the findings of this study are available from the corresponding author upon request.

## Abbreviations

XDD: Xijiao Dihuang Decoction
TCM: Traditional Chinese medicine
Nim: Nimodipine
MCAO: Middle cerebral artery occlusion
HE: Hematoxylin and eosin
CCA: Common carotid artery
ECA: External carotid artery
ICA: Internal carotid artery
MCA: Middle cerebral artery
SDS-PAGE: Sodium dodecyl sulfate-polyacrylamide gel electrophoresis
BPI: Base peak intensity
BDNF: Brain derived neurotrophic factor
GDNF: Glial cell line-derived neurotrophic factor
VEGF: Vascular endothelial growth factor
bFGF: Basic fibroblast growth factor.

## Additional Points

*Statement*. Our results in this study did not indicate that Bubali Cornu (water buffalo horn), component of animal origin, is not required or that an alternative or synthetic substitute could be used.
